# Supplementary material for: A Critical View of the Application of the APEX Software (Aqueous Photochemistry of Environmentally-Occurring Xenobiotics) to Predict Photoreaction Kinetics in Surface Freshwaters
Source: Molecules. 2019 Dec 18;25(1):9. doi: 10.3390/molecules25010009 (PMC7017383; doi:10.3390/molecules25010009)
Supplement: Supplementary file 1 [file molecules-25-00009-s001.zip › 3.2.4_gcc-4.4.0.zip!/3.2.4_gcc-4.4.0/3.2.4_gcc-4.4.0/doc/octave/html/interpreter/Handling-Errors.html]

Handling Errors - Untitled


Next: Handling Warnings,
Up: Errors and Warnings


---

### 12.1 Handling Errors

An error is something that occurs when a program is in a state where
it doesn't make sense to continue. An example is when a function is
called with too few input arguments. In this situation the function
should abort with an error message informing the user of the lacking
input arguments.

Since an error can occur during the evaluation of a program, it is
very convenient to be able to detect that an error occurred, so that
the error can be fixed. This is possible with the `try` statement
described in The `try` Statement.

- Raising Errors- Catching Errors
